# ASAS-NANP SYMPOSIUM: MATHEMATICAL MODELING IN ANIMAL NUTRITION: Modeling the dynamics of complex systems: incorporating AI and HPC into dynamic modeling

**DOI:** 10.1093/jas/skag310

**Published:** 2026-10-03

**Authors:** Ignacio J Martinez-Moyano

**Affiliations:** Decision and Infrastructure Sciences Division, Argonne National Laboratory, Lemont, IL 60439, United States; Consortium for Advanced Science and Engineering, The University of Chicago, Chicago, IL 60637, United States

**Keywords:** artificial intelligence, dynamic modeling, high-performance computing, modeling and simulation, system dynamics

## Abstract

In many areas of animal science, the questions are dominated by dynamic problems: outcomes evolve over time, adapt to interventions, and emerge from interacting biological, managerial, and institutional processes operating across temporal and spatial scales. These systems exhibit accumulation, feedback, nonlinearities, and time delays that can make intuitive reasoning unreliable, and that can cause well-intended policies to underperform or generate unanticipated consequences. Dynamic modeling and simulation provide a rigorous way to make such mechanisms explicit and to test whether observed patterns of behavior can arise endogenously from system structure. This paper positions system dynamics modeling as a foundational approach for feedback-driven explanation and policy design in animal systems. It also highlights agent-based modeling as a natural approach to capture heterogeneity, networks, and localized interactions in system behavior. The paper describes how artificial intelligence can strengthen dynamic modeling workflows by scaling evidence synthesis, supporting transparent and auditable structure development, and expanding feasible calibration and updating methods under uncertainty. Finally, the paper describes how high-performance computing enables ensemble-based inference and robust policy evaluation by making large-scale sensitivity analysis, uncertainty quantification, calibration, and scenario development and testing routine. Across these themes, the paper emphasizes methodological discipline: artificial intelligence and high-performance computing should augment—not replace—critical thinking, causal reasoning, rigorous structure development, thoughtful analysis, and transparent reporting. The combined use of dynamic modeling, artificial intelligence, and high-performance computing can help animal science build interpretable, testable, and updateable models that support learning and timely decision making in complex systems.

## Teaser

Dynamic modeling and simulation help animal-science researchers and decision makers understand how relevant elements in a system interact over time (eg farms, animals, diseases, regulations, and markets)—so interventions in such systems can be thoroughly understood and tested before they are implemented. By combining transparent simulation models with artificial intelligence and high-performance computing, this approach improves the ability to evaluate policies under uncertainty and reduce unanticipated consequences.

## Introduction

Animal science deals with dynamic problems. Dynamic problems evolve through time, adapt to interventions, and are the result of processes that operate at different temporal, spatial, and organizational scales. Biological systems accumulate and deplete resources (eg body reserves, pathogen load, immunity, and sources of energy), management systems allocate effort and capital under constraints, and markets and institutions respond to signals with delays. These interacting mechanisms create nonlinear responses, threshold effects, and time lags that make short-term improvements difficult to sustain and long-term consequences difficult to identify and anticipate. As a result, actions intended to correct problems in this domain may have limited effect, shift burdens within systems, or amplify the very outcomes they were meant to mitigate.

Modeling and simulation can provide a disciplined approach to making the dynamics at play explicit and to identifying and understanding the underlying system structure that creates such dynamics. In contrast to static or linear analyses, dynamic modeling is designed to identify and explicitly represent accumulation, feedback, nonlinearity, and delay—features that are central to animal systems, from within-animal physiology to herd-level behavior, and from epidemiology to supply-chain considerations. System dynamics (SD) offers one such approach (Forrester 1958, 1961; Sterman 2000; Martinez-Moyano and Richardson 2013; Martinez-Moyano 2018, 2023): SD represents systems as collections of interacting stocks and flows linked by information feedback and decision rules, enabling cause-and-effect level of analysis that allows the modeler to ask whether observed patterns can arise endogenously from system structure. In animal-production contexts, SD has been used as a model-based decision support approach where controlled experimentation is expensive or slow and where policy assessment requires exploring scenarios over long horizons. For example, Tedeschi et al. (2011) emphasized SD as a practical tool to develop management insights for animal production systems characterized by feedback, delays, nonlinear responses, and constraints that are difficult to study comprehensively in the field.

Dynamic modeling in animal science is also increasingly methodologically pluralistic. Agent-based modeling (ABM) (North and Macal 2007), discrete-event simulation, and hybrid approaches (Martinez-Moyano and Macal 2016) are frequently used alongside SD to capture heterogeneity among animals and stakeholders, discrete events, spatial interactions, and adaptive behavior. A recent *Journal of Animal Science* symposium paper highlights ABM’s value for livestock systems by enabling representation of individual agents, their attributes, and their adaptive responses—capabilities that are particularly relevant when aggregation masks critical behaviors or when interventions act through changes in decision rules rather than through direct biological mechanisms (Kaniyamattam and Tedeschi 2023). In practice, different modeling and simulation approaches can be complementary and should be used to answer the questions posed at the correct level of aggregation: SD can clarify dominant feedback structures and long-term behavior, while ABM can resolve fine-grained heterogeneity and localized interactions that influence those feedbacks.

Two technological developments make this a timely moment to articulate a general framework for dynamic modeling and simulation in animal science. First, data streams are expanding rapidly (eg precision livestock farming, animal-related digital infrastructures), creating new opportunities to create models and to connect such models to observations and to update state estimates and parameters more frequently. Recent reviews describe a broadening portfolio of artificial intelligence (AI) applications in livestock systems, including computer vision for identification and behavioral measurement and, more recently, large language models (LLMs) as interfaces for knowledge retrieval and decision support (Menezes et al. 2024). These AI capabilities can strengthen dynamic modeling workflows in three concrete ways. First, AI-assisted evidence synthesis—using natural language processing and large language models to extract candidate variables, relationships, and parameter estimates from text, interviews, and administrative records—can accelerate the construction of causal-loop diagrams and reduce the manual coding effort of conceptualization (Hosseinichimeh et al. 2024; Veldhuis et al. 2024). Second, machine-learning surrogates and feature-extraction techniques can summarize complex model outputs and identify regions of parameter space worth exploring, which in turn supports more targeted policy exploration (Akhavan and Jalali 2024). Third, modern Bayesian and amortized inference methods—including Hamiltonian Monte Carlo and simulation-based amortized Bayesian inference—make calibration of complex dynamic models computationally feasible at scales that would not have been practical a few years ago (Andrade and Duggan 2021; Rahmandad et al. 2025). In all three cases, the modeler remains responsible for biological plausibility, causal structure identification and simplification, and policy interpretability.

Beyond the expansion of data streams and AI capabilities, a second technological development is equally important: the computational demands of credible dynamic inference and robust policy exploration using ever-expanding datasets and model detail. Complex animal systems are uncertain: parameters vary across environments and genetics, interventions change behavior and incentives, and rare events can dominate outcomes. Addressing these realities typically requires large ensembles of simulation runs for sensitivity analysis, uncertainty quantification, and scenario stress-testing. High-performance computing (HPC)—together with modern software that uses efficient process parallelization—makes it feasible to perform such analyses at scales that were previously impractical or impossible, shifting emphasis from single-best-estimate trajectories to ensembles of plausible futures. Accordingly, this paper (1) summarizes core principles of feedback-driven dynamics relevant to animal science, (2) outlines how AI can support model development, calibration, and use without eroding interpretability, and (3) describes how HPC enables scalable uncertainty-aware inference and policy evaluation for complex animal systems.

## Modeling complex systems

System dynamics (SD) is a feedback-based modeling and simulation approach designed to explain and improve the behavior of complex systems over time. Its central claim is that the internal structure of the system gives rise to its characteristic patterns of behavior (eg growth, decline, oscillation, overshoot, collapse, and dynamic equilibrium). The internal systemic structure includes accumulations (stocks), their rates of change (flows), information feedback, boundedly rational decision rules, delays, and nonlinear relationships. SD emphasizes endogenous explanations of system behavior. Instead of attributing dynamics mainly to external shocks, SD seeks to identify, explain, and show how the system’s own internal structure generates much of its observed behavior—both desired outcomes and problematic ones (Forrester 1961; Richardson and Pugh 1981; Sterman 2000; Martinez-Moyano 2023).

The SD approach is especially useful in settings characterized by dynamic complexity—situations where cause and effect are distant in time and space; where local changes aimed at improving the system can generate remote problems; where policies produce unanticipated consequences and resistance; and where the interactions among individual actors in the system, institutions, and physical, psychological, sociological, and organizational constraints create counterintuitive results. Because complex systems are typically governed by feedback effects, include time delays, exhibit nonlinearities, and are history dependent, the SD approach is well suited to generate valuable insights into their inner workings and the way in which influences are transformed into results (Forrester 1961; Sterman 2000; Martinez-Moyano and Richardson 2013; Nicholson et al. 2019; Martinez-Moyano 2023). Complex systems features can—and in many cases do—render unaided human intuition unreliable: in complex systems, people systematically misperceive feedback and stock–flow relationships (Sterman 1989), misunderstand and overweigh short-term outcomes, fail to see the role that accumulations in the system have on system inertia, and underappreciate the time delays that make systems hard to control. SD addresses these limitations by taking an endogenous perspective, formalizing dynamic hypotheses, simulating policy implications, and using disciplined in-silico experimentation to improve models of the system, advance theory and policy, and inform real-world decisions.

## SD building blocks: feedback, stocks and flows, delays, and nonlinearity

Feedback is at the core of SD. A feedback loop exists when a change in a variable eventually feeds back to influence itself, either reinforcing the initial change (reinforcing—also called positive—loops) or counteracting it (balancing—also called negative—loops). Reinforcing loops generate growth (eg contagion, diffusion, cumulative advantage, learning-by-doing, and escalation). Balancing loops generate balance in the systems (eg goal seeking, regulation, stabilization, and constraint-driven limits). Real-world dynamics arise from the shifting dominance of multiple interacting loops over time (Sterman 2000, 2001).

Stocks and flows are the backbone of the internal structure of complex systems. Stocks represent accumulation (eg inventory, installed capacity, workforce, trust, capital, backlog, susceptible population, and pollutant concentration) while flows represent the rates that change—increase or decrease—such accumulations. A crucial implication is that stocks provide the systems with inertia and memory: stocks change only through flows and, consequently, the interventions that change flows can change the stock but only gradually and over time. There are few examples in which punctuated changes to accumulations can happen; in general, accumulations change gradually, and initial changes often trigger balancing loops that resist further change. In SD, these relationships are explicit in stock-and-flow maps, where sources/sinks represent boundary conditions when mechanisms outside the chosen boundary are not the focus of analysis (Martinez-Moyano 2018).

Time delays are another defining feature of complex systems. Time delays may be connected to physical characteristics of the system (eg construction and production lead times), informational characteristics (eg measurement, reporting, and perception lags), behavioral characteristics (eg learning, habit formation), or institutional characteristics (eg approval and coordination processes). Delays that are not adequately identified and understood tend to destabilize policies by allowing decision processes to react to information signals that can be premature or outdated. When coupled with nonlinearities within the system—capacity limits, saturation effects, thresholds, and diminishing returns—delays can exacerbate many of the patterns that are considered problematic and that SD aims to explain (eg overshoot-and-collapse, sustained oscillations).

## The SD modeling process

The SD modeling process is iterative by design (Sterman 2000; Martinez-Moyano and Richardson 2013): new understanding generated by simulation motivates revisions to problem definition, model boundary, and structure (see Figure 1). The recognition of the existence of a problem becomes the driving force of model building, and iteration is essential to refine both the model and the problem framing. In their work, Martinez-Moyano and Richardson (2013) synthesize expert-elicited best practices into a staged process that includes problem identification and definition, system conceptualization and dynamic hypothesis development, model formulation, testing and evaluation, policy design, and implementation-oriented learning strategies.

**Figure 1:**
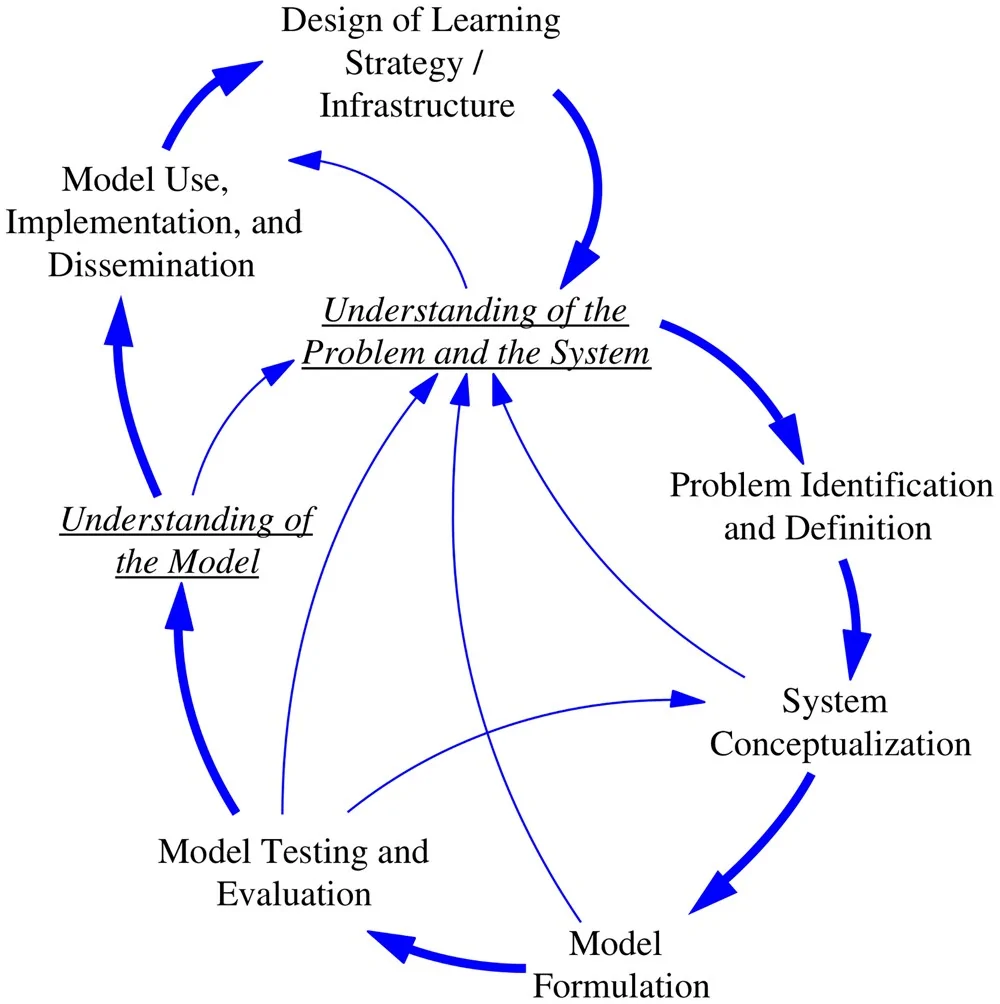
Overview of the system dynamics modeling process (see Martinez-Moyano and Richardson 2013, p. 108). Overview of the system dynamics modeling process, which is iterative by design. Understanding of the problem and the system (center) both drives and is updated by the surrounding activities, so that each pass through the process refines the model, the problem framing, and the resulting policy insight. Adapted from Martinez-Moyano and Richardson (2013).

### Problem identification and definition

SD begins with a clearly articulated problem, defined in dynamic terms. Practically, this means expressing the problem as a reference mode—an empirical or perceived pattern of behavior over time—and selecting a time horizon long enough to capture key dynamics, not just short-run fluctuations. Clarifying the purpose of the model is essential, because purpose shapes what belongs inside the boundary and what can be treated as external or exogenous for the specific question at hand.

### System conceptualization and dynamic hypothesis

In the conceptualization process, the system boundary and the candidate feedback structures responsible for the existence of the reference mode are identified and clarified in a dynamic hypothesis. A dynamic hypothesis is a concise, provisional explanation linking feedback structure to the behavior of interest. It is provisional because it must be operationalized, tested, refined, and verified as relevant for the problem at hand. At this stage, boundary selection is aimed at including the minimum set of interacting components whose feedback generates the problematic dynamics. The idea is to generate the simplest possible representation of the relevant elements of the system (the simplest model), but not simpler than that (following Occam’s razor, parsimony is the guiding principle). The SD preference for endogenous explanations of behavior follows directly: if key drivers are treated as exogenous without strong justification, important feedback pathways may be severed, weakening deep knowledge about the system, the problem under study, and policy insight.

### Model formulation

Formulation translates the dynamic hypothesis into an operational stock-and-flow model with explicit mathematical representation, decision rules, units, and parameters. A widely recommended practice is to start with a small, simulatable core model early in the process and to add detail iteratively—so the modeler can trace which new structures improve explanatory power and which introduce unnecessary complexity (Martinez-Moyano and Richardson 2013). Dimensional consistency and extreme-condition reasoning provide rigor from the start. Also, all equations should make sense within plausible ranges (eg if the number of cows in the model is zero, milk production must be zero). These practices protect against formulation errors that can invalidate policy conclusions, lower confidence in model results, and obscure the value of the modeling exercise.

### Testing and confidence building

In SD, the mechanisms to gain confidence in results are built into the iterative process; they are not an after-the-fact element added at the end of modeling. Confidence is built through a portfolio of structural, behavioral, and policy-oriented tests that are applied repeatedly during model development (Forrester and Senge 1980; Barlas 1996; Sterman 2000). Common tests include boundary adequacy, dimensional consistency, extreme conditions, parameter plausibility, behavior reproduction, sensitivity analysis, and checks for numerical integration error. Contemporary SD practice also emphasizes transparency in reporting results, documenting assumptions, equations, and tests so that others can inspect, critique, and reproduce results (Martinez-Moyano 2012; Rahmandad and Sterman 2012).

### Policy design, learning environments, and implementation

Once the model can reproduce the reference modes and pass relevant structural and behavioral tests, it becomes a laboratory for policy testing and design. The SD approach, by focusing on how the feedback structure generates dynamic behavior, allows for in-depth understanding and examination of consequences of changes in assumptions and environmental conditions. Additionally, where feasible, the developed models can support interactive learning environments—management flight simulators and participatory modeling processes—that help stakeholders experience the likely consequences of their decisions under realistic feedback and delay conditions.

### Choosing architecture and level of aggregation

Modern SD practice increasingly engages questions of model architecture and scale. Sterman (2018) argues that despite vastly improved computational capabilities, the development of fully comprehensive models remains impossible because constraints on attention, data, purpose, and interpretability are always present. The appropriate level of aggregation of a model depends on purpose. Aggregated compartment models can be effective for capturing broad feedback dynamics, while disaggregation may be necessary to represent heterogeneity that alters behavior. This perspective supports hybrid and multimethod modeling when the purpose of the modeling effort calls for it. In this sense, SD is not competing with other approaches (eg agent-based modeling, discrete-event simulation, and econometrics) but inviting modelers to think about which architecture best represents the feedback mechanisms and decision rules relevant to the problem—at the level of detail required to answer the guiding question(s) of interest. In complex systems research, this fitness-for-purpose condition is always more useful than debates over methodological purity.

### Transparency, documentation, and reproducibility

In SD, transparency is considered a methodological requirement. Transparency is defined as making the formulation, data, assumptions, and results salient and readily inspectable, supporting confidence building and scientific reproducibility (Martinez-Moyano 2012). Documentation is also central to effective stakeholder engagement, as decision makers are more likely to trust and use models when they can understand what drives the results and how assumptions influence conclusions. Tools that automate documentation and assessment can help operationalize transparency: they lower the effort needed to produce thorough documentation, improve reviewability, reduce hidden errors, and support cumulative science (Martinez-Moyano 2024).

## What SD contributes to modeling complex systems

System dynamics contributes an explanation-centered approach that treats problematic behavior as an emergent consequence of endogenous feedback structure (Martinez-Moyano 2018). The method’s core elements—reinforcing and balancing feedback, stocks and flows, delays, and nonlinearity—provide a compact nomenclature for representing complex systems and associated dynamics, while the model building process promotes iterative refinement, explicit testing, and stakeholder learning (Martinez-Moyano and Richardson 2013; Sterman 2018; Martinez-Moyano 2023; Richardson 2025).

## Incorporating AI into dynamic modeling

As SD and related dynamic modeling approaches move into data-rich domains, two recurring issues have become more salient: converting heterogeneous evidence (text, interviews, field notes, administrative records, and sensor streams) into explicit, auditable model structure, and calibrating and updating models when uncertainty is high and parameter spaces are large (Akhavan and Jalali 2024). AI methods can be integrated at multiple stages of the SD modeling process (see Figure 1, introduced above). During problem identification and conceptualization, natural language processing and large language models can help synthesize literature, extract candidate variables and links from interviews and texts, and produce initial causal-loop diagrams for expert review (Hosseinichimeh et al. 2024; Veldhuis et al. 2024). During model formulation, AI-assisted tools can suggest functional forms for decision rules, help identify dimensional inconsistencies, and draft documentation for review by the modeler (Akhavan and Jalali 2024). During calibration, Bayesian workflows—including Hamiltonian Monte Carlo and amortized Bayesian inference—enable principled estimation of parameter uncertainty for complex dynamic models (Andrade and Duggan 2021; Rahmandad et al. 2025). During testing and policy design, machine-learning surrogates and meta-models can accelerate sensitivity analysis and the exploration of large policy spaces. Across these stages, recent work frames AI as a complementary set of methods that can accelerate evidence extraction, expand feasible calibration processes, and support reproducible sensitivity and policy exploration—while leaving causal reasoning, validation, and interpretation as core responsibilities of the modeler (Andrade and Duggan 2021; Rahmandad et al. 2025). The next two subsections discuss in greater detail (a) AI in support of model building (ie structure development from data and text) and (b) AI in support of calibration and updating.

## AI in support of model building

One challenge in SD is the identification of relevant causal factors and feedback mechanisms in a system of interest, relative to the problem of interest and to the purpose of the model. A second, closely related challenge is how to use the available data—mental models, observational records, historical series, and textual sources—especially at scale, to identify and characterize causal structures without losing the interpretability that makes SD powerful (ie identifying and discovering causal structure from data). Some approaches have been proposed to infer causal-loop-diagram-like structures from data, aiming to generate testable hypotheses about feedback mechanisms that can then be scrutinized by domain experts and confronted with additional evidence (Abdelbari and Shafi, 2017). These methods are most defensible when treated as hypothesis generators rather than automated model builders.

Text is an especially important evidence source in animal systems and other complex environments (eg producer decision making, biosecurity practices, adoption of technologies, extension guidance, and after-action reviews). Natural language processing can identify candidate causal statements at scale and prioritize material for deeper coding, while also highlighting the fact that linguistic causality is not always the same as structural relevance for dynamic models (Veldhuis et al. 2024). Expert scrutiny is still needed to clarify the adequacy and usefulness of algorithmic findings.

Work in SD has aimed at increasing the effectiveness of the use of textual data in the process of identifying and developing causal structures (Martinez-Moyano and Collier 2015; Hosseinichimeh et al. 2024). Recent work has operationalized this idea using SD bots to extract candidate variables and links from textual data to construct causal loop diagrams (Hosseinichimeh et al. 2024). In applications related to animal science, AI-based tools have the potential to be used to accelerate model building especially in cases where diverse stakeholders bring different vocabularies and mental models, by producing initial sets of causal relationships that teams can correct, refine, and validate (Vennix 1996; Rouwette et al. 2004).

Large language models (LLMs) add a newer layer of AI-assisted capability to the model building process. LLMs can help summarize evidence, identify candidate variables, draft documentation, and propose formulations of decision rules. LLMs and other AI-assisted tools should augment rather than replace critical thinking in the model formulation process and in the subsequent interpretation of model results (Akhavan and Jalali 2024). To be useful in modeling, AI-assisted tools should be able to leave an audit trail (eg candidate causal statements with citations to source text) and generate reproducible results.

## AI in support of calibration and updating

Beyond structure development, AI-assisted methods can strengthen calibration and updating, and it is worth distinguishing the different families of methods involved. Classical Bayesian workflows for SD models—such as the Hamiltonian Monte Carlo approach demonstrated by Andrade and Duggan (2021)—represent parameter uncertainty through intentionally chosen priors and sample from joint posterior distributions that have no closed-form expression. These methods are statistically principled and not, strictly speaking, AI in the modern sense; they pre-date current AI developments by decades, even though they often share computational infrastructure with modern machine learning. What is genuinely new is the use of deep learning and other AI techniques to make such Bayesian inference scalable for complex dynamic models. A major step in this direction is amortized Bayesian inference, where neural networks are trained on synthetic simulations generated from the SD model to learn an inverse mapping from observed time series to parameter posteriors. This likelihood-free approach enables near-instant posterior estimation once training is complete, making it feasible to incorporate calibration and uncertainty analysis into iterative model development (Rahmandad et al. 2025). Other AI-assisted calibration approaches that go beyond classical Bayesian methods include (a) machine-learning surrogates or emulators—Gaussian processes, polynomial chaos expansions, or neural networks trained on simulation outputs—that approximate the dynamic model and accelerate parameter search and sensitivity analysis; (b) optimization-based calibration using evolutionary algorithms, simulated annealing, or reinforcement learning that can navigate non-convex, high-dimensional parameter spaces; and (c) LLM-assisted scaffolding of calibration workflows, in which language models help draft calibration code, propose plausible parameter ranges from the literature, and document decisions for review (Akhavan and Jalali 2024). These AI-assisted methods are complements to, not replacements for, classical Bayesian workflows: each has different assumptions, computational profiles, and validation requirements, and the choice among them should be driven by the problem at hand and by what the modeler needs to learn from the calibration exercise.

AI-assisted methods have a high potential to introduce unanticipated and novel failure modes in the model building process if they are used to automate steps that require careful critical thinking and domain judgment. Structure-learning approaches may generate spurious links, and NLP and LLM outputs suffer from biases and may not generate defensible causal mechanisms. Recent empirical work in a closely related applied domain illustrates this risk: in a comparison of ecological and fisheries models produced by agentic AI fitted to ecological data, the resulting models—although technically functional—diverged substantially in their management and policy implications, underscoring the need for calibrated models that produce results for the right reasons (Brown et al. 2026). Consequently, AI integration should be subordinated to SD’s established quality controls in model development, including the explicit articulation of purpose, transparent mapping from evidence to structure, rigorous tests for model behavior and robustness, stakeholder review of assumptions, and a clear understanding and communication of the effects of uncertainty on model specification, calibration, and results. When AI-assisted tools are used in the model building process, the process should preserve provenance (what data or text was analyzed), traceability (how outputs informed the model), and reproducibility (code, prompts, and model versions). Recent guidance on responsible use of generative AI in simulation research can provide a useful framework in the model building process (see Akhavan and Jalali 2024).

Many AI-assisted workflows are computationally demanding, especially when they require large synthetic training datasets or large-scale iterative policy exploration. The next section therefore examines how HPC enables scalable, uncertainty-aware simulation experiments that make these workflows practical in complex systems analysis and applied animal-science settings.

## Leveraging high-performance computing in dynamic modeling

High-performance computing (HPC) has the potential to help in dynamic modeling by turning computationally intensive workflows into routine parts of model development and application. In animal science, dynamic models frequently connect biological processes (growth, metabolism, reproduction, and immunity), population dynamics (herd demography, contact networks, and movement), and human and organizational decision making (management practices, biosecurity behavior, and market responses) across multiple interacting time scales. System dynamics (SD) modeling provides causal, feedback-based explanations of such behavior, while agent-based modeling (ABM) represents heterogeneity and interaction mechanisms explicitly. Both approaches generate their strongest insights not from a single simulation, but from repeated simulation across uncertainty ranges, alternative assumptions, and policy levers. HPC is an enabling infrastructure for model development and use as it supports large simulation ensembles needed for rigorous analysis, sensitivity, uncertainty quantification, calibration, and robust intervention testing, and it shortens iteration cycles when models must be updated as new data arrive (Nicholson et al. 2019; Ozik et al. 2021).

## What HPC, in practice, enables in dynamic modeling

In dealing with real-world problems using dynamic modeling, many times a limiting factor is not the ability to run a model once, but the ability to run it enough times to learn reliably. HPC increases the scale and speed of four key activities that can determine the usefulness of dynamic models for decision support.

First, HPC enables ensemble simulation at scale. Large ensembles are essential when outcomes are sensitive to uncertain inputs, stochastic realizations, or alternative structures. Ensembles support reporting distributions of outcomes rather than single trajectories and enable stress testing of policies under wide-ranging conditions (ensembles of 1,000–10,000 runs are commonly reported for global sensitivity analysis).

Second, HPC supports systematic sensitivity analysis. High-dimensional sensitivity methods often require hundreds to thousands of runs, and even efficient designs remain computationally demanding for dynamic outcomes observed over time (Clemson et al. 1995; Kleijnen 1995; Ford and Flynn 2005; Jadun et al. 2017).

Third, HPC makes uncertainty-aware calibration feasible for more complex models. Bayesian calibration and related workflows often require many model evaluations (eg multiple chains and repeated updates), and as model size and parameter dimensionality grow these requirements scale rapidly. Several HPC-enabled techniques make this tractable. Parallel Markov Chain Monte Carlo schemes distribute multiple independent chains across compute nodes, which both shortens wall-clock time and supports convergence diagnostics across replicates (Andrade and Duggan 2021). Likelihood-free and amortized inference methods—in which neural networks are trained on large libraries of synthetic simulations generated in parallel on HPC resources—learn an inverse mapping from observed time series to parameter posteriors and enable near-instant inference once trained (Rahmandad et al. 2025). Surrogate or emulator models (Gaussian processes, polynomial chaos expansions, and neural-network surrogates) trained on parallel simulation ensembles can approximate the response surface of an expensive dynamic model and dramatically accelerate calibration and sensitivity analysis. For agent-based and hybrid models, ensemble Kalman filtering, sequential Monte Carlo, and particle filtering implementations distributed across compute nodes provide online state and parameter updating as new data arrive (Ozik et al. 2021). Together, these methods convert calibration from a one-off, often heuristic step into a repeatable, uncertainty-aware component of the modeling pipeline that scales with model complexity.

Fourth, HPC enables rapid, repeatable pipelines for scenario and policy exploration by scaling the execution of structured scenario libraries and supporting storage and reuse of large simulation outputs for downstream analysis (Peterson et al. 2019). In typical implementations, this is realized through several complementary technologies. Workflow orchestration systems such as Extreme-scale Model Exploration with Swift (EMEWS) coordinate large ensembles of simulation runs across heterogeneous compute resources and enable adaptive, model-in-the-loop experimentation in which sampling strategies are updated as runs complete (Ozik et al. 2021). General-purpose workflow managers (eg Snakemake, Nextflow, and Dask) and cluster schedulers (eg SLURM) play the same role for less specialized workflows. Parallel simulation frameworks such as Repast HPC and Repast4Py enable single, large ABMs to be distributed across compute nodes, making it possible to simulate millions of agents with explicit interaction networks (Collier and North 2013; Collier and Ozik 2022). Containerization (Docker, Singularity/Apptainer) and reproducible environment management ensure that the same model and software stack can run on a laptop, a campus cluster, or a leadership-class supercomputer—reducing the friction of moving from development to production. Outputs are typically stored in self-describing, parallel-friendly formats (eg HDF5, Parquet) and queried with parallel analytics tools, which makes it practical to revisit and re-analyze archived ensembles as new questions arise. Linking these components into a single, version-controlled pipeline makes the same analysis re-runnable when new data arrive or when a reviewer requests a different scenario—a core requirement for iterative SD practice and cumulative science.

## HPC in dynamic modeling

In SD, HPC is most commonly realized as high-throughput execution of many simulations rather than parallelization of a single run. This is the result of common SD practice in which learning depends on exploring parameter uncertainty, policy alternatives, and structural hypotheses. Experimental-design ideas such as Latin hypercube sampling and regression-based metamodeling help keep sensitivity analysis tractable while providing broad coverage of multidimensional parameter spaces (Clemson et al. 1995; Kleijnen 1995). Subsequent work operationalized statistical screening methods that can identify influential parameters among many uncertain inputs, indicating when different mechanisms dominate system behavior (Ford and Flynn 2005; Taylor et al. 2010). Variance-based methods quantify not only main effects but also interactions among uncertain inputs—an important feature for animal systems, where intervention outcomes often depend on interacting biological and behavioral processes. This motivates the use of HPC to make such analyses routine (Jadun et al. 2017).

HPC also supports SD projects at scale by enabling reproducible scenario pipelines and curated output datasets. Large-scale modeling efforts show how thousands of simulations can be generated from structured scenarios, executed at scale, and stored for analysis and reuse, supporting collaborative, iterative modeling with explicit provenance (Peterson et al. 2019). Operationally, a large-scale SD pipeline typically combines four elements: (a) a versioned model repository with a scriptable simulation engine (eg Vensim DSS in batch mode, Stella’s command-line tools, or open-source engines such as PySD or BPTK-Py) so that runs can be launched programmatically; (b) a scenario-definition layer that encodes parameter sweeps, policy levers, and assumption variants in a machine-readable form (eg YAML, JSON, or database tables); (c) a workflow manager that dispatches runs to compute resources, tracks provenance, and handles failures and retries; and (d) a curated output repository that stores simulation results with metadata sufficient to reproduce each run (model version, parameter set, random seed, and software environment). The Biomass Scenario Model project (Peterson et al. 2019) is a concrete example of this pattern in an energy and bioeconomy context: thousands of simulations were generated, executed, archived, and re-analyzed across multiple research questions. The same pattern is directly applicable to animal-science problems—for example, exploring herd-management policies under climate and market uncertainty, evaluating antimicrobial-stewardship interventions across regional supply chains, or analyzing welfare-transition pathways under alternative regulatory regimes—where the ability to re-run a coherent scenario library as new data or new policy questions emerge is what converts a one-time modeling study into a sustained decision-support capability.

Highly disaggregated models (eg agent-based models) can benefit from HPC resources for at least two reasons beyond those common to aggregate modeling such as SD modeling. First, ABMs often represent large, heterogeneous populations and explicit interaction networks (eg animals, farms, workers, movements, and markets). When the number of agents, places, and contacts grows, single-machine simulation can become limited by both computation and memory. Second, ABMs commonly include stochasticity at various levels of their design to capture some of the aspects of the heterogeneity that they try to represent, which means robust inference typically requires many replicates for each parameter set and policy scenario. In the case of ABMs, HPC is used to enable the efficient execution of larger, highly detailed models via parallel and distributed execution, and to enable the execution of ensemble experiments for uncertainty analysis and policy testing. Parallel ABM frameworks and workflow control tools provide concrete pathways for scaling (Collier and North 2013; Collier and Ozik 2022). Operational epidemic modeling provides a vivid illustration of the potential. A population data-driven HPC workflow for epidemic modeling describes how detailed agent-based simulations can be coupled with large-scale computation to support rapid scenario analysis and learning under time constraints (Ozik et al. 2021; Hotton et al. 2022).

For animal-science applications, HPC adds the most value when it strengthens the defensibility of conclusions rather than simply increasing model size. Three implications are especially salient. First, HPC supports model accountability through routine uncertainty and sensitivity analysis, enabling uncertainty bands, interaction effects, and robustness claims to be reported as standard outputs. Second, HPC enables faster learning cycles in participatory and applied settings by reducing the friction of rerunning ensembles as stakeholders revise assumptions or new evidence becomes available. Third, HPC provides the computational backbone for AI-assisted workflows discussed in the prior section (eg surrogate modeling, likelihood-free inference, and algorithm-guided policy exploration).

The use of HPC resources is not a substitute for careful and disciplined modeling practice, well-developed model structure, careful documentation and verification, and critical and conscientious interpretation. The value of HPC in dynamic modeling is that it makes disciplined computationally intensive practices—ensemble testing, system-wide sensitivity analysis, uncertainty-aware calibration, and structured scenario development and libraries—practical at the scale required for modern data-rich complex dynamic systems.

## Conclusions and the road ahead

Dynamic problems in animal science—such as disease control, antimicrobial stewardship, welfare transitions, environmental impacts, and supply-chain resilience—are shaped by feedback, delays, nonlinearities, heterogeneity, and adaptive behavior. SD addresses this dynamic complexity by focusing on endogenous explanations of behavior over time (Forrester 1961; Richardson and Pugh 1981; Sterman 2000). As data streams and computational requirements grow, two enabling capabilities become central: AI, which strengthens evidence-to-structure and evidence-to-parameter workflows, and HPC, which makes ensemble-based inference and robust policy evaluation practical at scale.

Taken together, dynamic modeling, AI, and HPC form a complementary triad. Aggregated dynamic modeling such as SD provides causal grammar for accumulation, feedback, and endogeneity; while disaggregated approaches such as ABM provide the capacity to represent heterogeneity in the system; AI supports scalable evidence synthesis and calibration; and HPC supplies the throughput and workflow infrastructure for large ensembles, global sensitivity, uncertainty quantification, and scalable exploration. The opportunity is not simply to build larger models, but to build more credible and more useful models—models that can be inspected, tested, updated, and communicated in ways that support cumulative science and responsible decision making (Rahmandad and Sterman 2012; Nicholson et al. 2019).

It is crucial to emphasize that the same tools that can accelerate the modeling process can also accelerate—and obscure—error generation. AI-assisted structure extraction can produce plausible but spurious causal stories (Veldhuis et al. 2024) and large HPC-enabled ensembles can generate false confidence if calibration is attempted with weakly identified parameters or if structural uncertainty is ignored. Put differently, the ability of AI-assisted tools to produce a working, well-fitting model is not evidence that the model is correct: a model that runs and fits historical data may still misrepresent the causal structure of the system and therefore yield misleading policy conclusions. Expert oversight, structural testing, and transparent reporting of uncertainty remain essential safeguards. AI and HPC should be subordinated to the rigor of methodological disciplines that make dynamic modeling credible—clear articulation of purpose; boundary adequacy; dimensional consistency; extreme-condition reasoning; behavior reproduction where appropriate; comprehensive documentation; consistent and transparent reporting of experiments, assumptions, and uncertainty (Forrester 1961; Barlas 1996; Sterman 2000; Martinez-Moyano 2012; Rahmandad and Sterman 2012). In animal-science contexts, these guardrails are also ethical and practical necessities. Models can influence decisions affecting animal welfare, producer livelihoods, and public health. Transparency therefore includes not only equations and code, but also explicit documentation of value tradeoffs, data limitations, and how uncertainty affects recommended actions.

Progress will be accelerated by five priorities:

1. *Fit-for-purpose model architecture and principled hybridization*. Model architecture should be chosen based on the decision question, not on methodological preference.
2. Evidence-to-model pipelines that preserve provenance and traceability. AI-assisted workflows should be developed as traceable pipelines rather than as opaque automation.
3. *Routine uncertainty analysis as a reporting expectation*. Dynamic models should report uncertainty as a first-class result, not as an optional add-on.
4. *Reproducible workflows and shared modeling assets*. HPC-enabled modeling benefits from workflow-centric practices that make analyses re-runnable and shareable.
5. *Investments in training and stakeholder-centered deployment*. Realizing the potential of SD, AI, and HPC requires investment in training and collaborative practice.

With these foundations, dynamic modeling can play a larger role in helping animal science anticipate undesired consequences, design robust interventions, and support sustainable, welfare-conscious outcomes in complex animal systems.

## Abbreviations

AI: artificial intelligence
HPC: high-performance computing
LLM: large language model
NLP: natural language processing
SD: system dynamics
